# Dietary patterns and associated risk factors among school age children in urban Ghana

**DOI:** 10.1186/s40795-018-0230-2

**Published:** 2018-05-10

**Authors:** Deda Ogum Alangea, Richmond N. Aryeetey, Heewon L. Gray, Amos K. Laar, Richard M. K. Adanu

**Affiliations:** 1 Department of Population, Family and Reproductive Health, School of Public Health, University of Ghana, Legon, Ghana; 2 Department of Community and Family Health, College of Public Health, University of South Florida, Tampa, USA

**Keywords:** Dietary patterns, Dietary behaviour, School-age children, Ghana, Overweight, Energy-dense

## Abstract

**Background:**

Understanding dietary patterns in the study of diet-disease relationships is crucial for designing dietary behaviour interventions. This study aimed to determine associations between dietary patterns and background characteristics among school age children (9–15 years) in Ghana.

**Methods:**

A cross-sectional sample of 487 urban-dwelling children age 9–15 years was recruited using simple random sampling from 24 schools (12 private and 12 public) in the Ga-East Municipality in Southern Ghana. A 7-day food frequency questionnaire was used to record children’s consumption of over 100 unique food items. Principal component analyses based on 14 food groups was used to describe emerging dietary patterns (DP). BMI-for-age z-scores segregated by sex were derived using WHO Anthro plus software. Linear regression was used to test associations between ‘diet factor’ scores, and weight status controlling for age.

**Results:**

Four DPs were identified that explained 53.2% of variation in the diets of children: (1) energy dense; (2) starchy root staple and vegetables; (3) cereal-grain staples and poultry; and (4) fish & seafoods. Energy dense DP characterised by processed meat, fried foods, and sugary foods was associated with child overweight/obese status after controlling for age, sex, SES and school type [F(5, 484) = 6.868, *p* < 0.001]. Starchy root with vegetable DP was negatively associated with overweight/obese status, private school attendance and higher SES after controlling for age at bivariate level. However, relationship between ‘starchy root staples and vegetables’ DP and overweight/obese status lost significance after controlling for other covariates.

**Conclusion:**

Our data identified energy-dense dietary pattern to be significantly associated with childhood overweight and obesity. Targeted dietary messages are required to address energy-dense dietary patterns among school-age children.

**Electronic supplementary material:**

The online version of this article (10.1186/s40795-018-0230-2) contains supplementary material, which is available to authorized users.

## Background

Nations worldwide are experiencing the nutrition transition which is characterised by rapid urbanisation, industrialisation, trade expansions, changing diets, decreased physical activity and a consequent rise in non-communicable diseases [1–4]. Although most West African countries are in the early stages of this transition, Ghana and other countries like Cape Verde and Senegal are at latter stages [5]. The characteristic changes in diets from traditional foods (less saturated fats and sugars, more whole grains, fruits and vegetables) to more ‘westernised diets’ (high in saturated fats, protein, refined carbohydrates, simple sugars and salt) and frequent consumption of fast foods has been observed in non-western societies [5–8].

The changing patterns of food consumption in Ghana has been well documented by Goody and Goody [9]. The major staples grown and consumed in most parts of West Africa, Ghana included are maize, yams, plantains and cassava [9], which are often eaten with soups or stews or used in making porridges or gruels. Over the years, the introduction of rice and other cereal cultivation in most parts of Ghana resulted in increased grain consumption. Typical Ghanaian meals are giving way to continental westernized/Asian alternatives due to inconvenient cooking methods. Plus, these meals need special handling when not eaten immediately. Some local foods with longer shelf-life, like ‘*gari*’ (grated, roasted cassava), ‘^1^*kenkey*’ and ‘^2^*banku*’ are inconvenient for busy commuting urban dwellers due to the accompanying stews/soups. This situation is creating increased interest among urban dwellers for processed pre-packaged foods such as sandwiches, biscuits, cheese, noodles, etc. Demand for such foods is also driven by prestige particularly among the middle and high income populations [9]. These unhealthy food options are actually less expensive per calorie compared to healthier options like lean meats, fish, fresh fruits and vegetables [10].

Since 1980, the world has witnessed an ‘obesity epidemic’ both among adults and children [3]. The number of obese children and adolescents (5–19 years) has risen from 11 million in 1975 to 124 million in 2016, a tenfold increase [11]. There is evidence that overweight and obesity prevalence is accelerating in LMICs including Ghana [12]. Although there is no nationally representative data on weight status of school-age children, prior studies have reported overweight/obesity prevalence between 7.8 and 25.9% [13–19] in urban Ghana. It is therefore crucial to ‘steer the ongoing nutrition transition towards a ‘more positive direction’ [7] in Ghana; which cannot be achieved in the absence of environmental modifications that encourage and sustain healthier lifestyle changes [20, 21].

Sub-optimal diets have been reported among school-age children and adolescents in developing countries including Ghana [22, 23]. While measurement of actual intakes of nutrients and calories is important, the single-nutrient approach to understanding diet-disease relationships is limited in its ability to account for the complexity of nutrient interactions in free-living individuals [24]. Empirical assessment of dietary patterns of entire diets, therefore, is useful to unravel complex diet-disease relationships. Dietary patterns are also easier for the lay public to relate with and to modify for better health outcomes compared to using nutrients or individual foods. It is therefore important that dietary patterns of school-age children be assessed to aid intervention design and delivery. The promotion of healthy dietary habits in childhood and adolescence would be necessary to prevent diet-related diseases. Intervening at the basic school level is crucial because adoption of unhealthy dietary habits tend to worsen with age in adolescence [25]. Optimal dietary habits in childhood is crucial for the prevention of diet-related diseases in populations since its effects transcends childhood [26]. Benefits of optimal diets include reduced risk of conditions such as anaemia, dental caries, obesity, eating disorders and non-communicable diseases such as cancers, type 2 diabetes, and cardiovascular diseases [27–32].

It is a public health priority globally to identify optimal diets for preventing chronic diseases [33]. Yet, very few studies have assessed empirical dietary patterns among Ghanaians [34, 35] with no published evidence on typical dietary patterns among Ghanaian school-age children. Existing studies have focused on meal consumption patterns or consumption of specific food groups and not an empirical description of patterns in dietary components [23, 36–38]. Dietary patterns in this study is refers to combinations of foods and drinks and their consumption frequencies observed in the population. To the best of our knowledge, this is the first study in Ghana to assess the dietary patterns of school age children in Urban Ghana. The objective of this paper is to empirically assess dietary patterns of school-age children 9–15 years and investigate their relationship with a priori determined child background characteristics (age, sex, socio-economic status, school type and weight status) in urban Ghana.

## Methods

### Study design, population and setting

A cross-sectional design was used to assess the dietary behaviour and food consumption patterns of basic school pupils aged 9–15 years. Participants were enrolled from both private (*n* = 12) and public (n = 12) schools in the Ga-East Municipality of the Greater Accra Region of Ghana. Participating schools had both primary and Junior high school departments. The Ga-East Municipality is one of 16 administrative districts of the Greater Accra Region which contains the nation’s capital, Accra.

### Sampling procedure and technique

The ability of children to adequately identify foods and recall dietary information is age-dependent. Thus, the minimum age for participating pupils was 9 years in accordance with recommendations regarding age-appropriateness of available dietary assessment methods [39]. The minimum sample size of 448 estimated was based on a pupil population of 50,200; expected frequency of obesity of 10%, assuming a least expected prevalence of 7% and a non-consent rate of 15%. A sample proportional to the size of each school was selected to make up a total sample size of 500 pupils. A total of 487 pupils eventually participated in the dietary assessment.

The full list of basic schools within the district was obtained from the Ghana Education Service (GES). Simple random sampling (balloting technique) was used to select 12 private and 12 public schools from a total of 80 (47 public and 37 private) registered basic schools (having both primary and Junior High School (JHS)) in the municipality. Within each school, balloting without replacement was used to select eligible pupils: Ballot bowls (separate coloured for boys and girls) containing pre-labelled (‘yes’ or ‘No’) ballot papers equivalent to the number of pupils on roll for specific research day were moved round primary classes 4 to JHS 3 by research assistants for pupils to pick. Selected pupils were given a written informed consent form to obtain parental consent. Completed consent forms were submitted to class teachers and subsequently collected by research team. Pupils who received parental consent also needed to complete an assent form to indicate child’s willingness to participate. As part of the assent process, the objectives, risks/benefits, costs and procedures of the study were explained to the children in their preferred language (English, Twi or Ga).

### Study measures and data collection procedures

#### Demographic characteristics

Child’s age in completed years; type of school (public versus private), educational level completed by mother/caregiver; residential status; and household ownership of assets (car, refrigerator/freezer, television, DVD/VCD player, computer, gas/electric stove and air conditioners).

#### Food frequency and dietary patterns

A 7-day Food frequency questionnaire (FFQ) (see Additional file 1) was used to record usual consumption data on a range of foods (about 100 unique food items). This FFQ was designed specifically for this study group and used in an earlier study among a larger population of Ghanaian school-age children [40]. Consumption of food items/sub-groups were indicated as the summation of the number of times each constituent food item(s) were consumed within the past 7 days. Factor scores for each identified pattern from PCA were saved for use in liner regression procedures.

#### Height and weight

Standing height was measured using the Charder HM200P portable stadiometer (Charder Electronic co., Ltd., Taichung, Taiwan) to the 0.1 cm. Body weight was measured with a portable Seca electronic weighing scale (SECA, Hamburg, Germany) to the nearest 0.1 kg.

Twelve research assistants were trained on how to administer the questionnaires and collect nutrition data as well as observe ethical procedures regarding research involving children. The study tools were pre-tested in two non-participating schools that had both primary and JHS departments within the school district. Study tools were interviewer-administered and lasted between 30 min and 1 h.

The research assistants probed for daily and weekly consumption as well as mixed meal options to help children with recall. Additionally, pupils were encouraged to indicate if they consumed foods that were not identified in the list of foods provided.

Measurement of weight (kg) and standing height (cm) was carried out following standard procedures for both weight and height measurements adapted from Shorr [41]. All measurements were taken at school with participants wearing their usual school uniform but without foot wear, socks, watches, and items in pocket or any heavy clothing like jacket or sweater. Interference of complex hairstyles with height measurements were reduced by encouraging participants to let hair down when possible and or by firmly pushing the head piece of height rod to compress hair. All anthropometric measurements were taken in duplicate. The same research assistants who administered the questionnaires were trained to take weight and height measurements of participants.

### Data analysis

Data were entered using a Microsoft access (2007 version) database, with activated checks to reduce errors. IBM SPSS version 20 software was used for data analysis. Frequency distributions were summarized in tables for categorical demographic characteristics. Arithmetic mean was determined for respondent age. Preliminary tests were carried out on data to check for normality/distribution (z- scores for outliers, Kolmogorov Smirnov test, Q-Q plots) and also reliability test (Cronbach’s alpha test statistic) performed on items included in Principal Component analyses (PCA).

Age (years) of respondents was grouped into the following categories: 9 to 10 years, 11 to 12 years and 13 to 15 years age groups); sex of pupil (either male or female); school administration type and household socio-economic status (SES) of respondents. Household SES was ascertained using principal component analysis. The factor score based on ownership of 8 items (car/ vehicle, house, television, refrigerator, gas/electric cooker, DVD/VCR/video player, air conditioner, and satellite dish) was used to group respondents into rich, middle and poor households [42]. Respondents were classified as overweight/obese or non-overweight/obese based on their BMI-for-age and sex (BMA) z-score. Overweight and obesity was defined as BMI for age-and –sex z-score (BMA) > 1 SD and ≥ 2 SD respectively; normal (non-obese/overweight) was defined as BMA ≤1 SD [43]. For the purposes of this study, pre-obese and obese groups were considered together in one group known as “overweight” and used for tests of associations.

Dietary patterns were assessed using PCA based on data collected with FFQ. In order to ensure that > 5% of respondents consumed each food item included in PCA, food items were categorized into several sub-groups based on shared nutritional value/significance. Sub-groups of foods (see Additional file 2) created for PCA are: processed meats; spreads and toppings; fried foods; cocoa beverages and Dairy; high caloric foods; poultry and eggs; starchy roots& tubers; sugar sweetened beverages; fruits and fruit juices; vegetables and soups; meats (all non-poultry meats); cereals/grains; legumes & nuts; and fish and sea foods. Thus, a total of 14 items were subjected to PCA in order to identify foods highly correlated with each other and possibly combined to describe the major patterns in respondent diets. PCA used orthogonal rotation (varimax) and number of components determined by Kaiser’s stopping rule (factors with Eigen values over 1.0 are considered in analysis) (Kaiser [44]) and examination of scree plots. Varimax rotation was used to maximize the factor loadings onto each component to allow ease of interpretation of patterns. Patterns were defined based on factor loadings > 0.4 and < − 0.4. In order to assess suitability of data for factor analysis, acceptable cut-offs for the Kaiser-Meyer-Olken (KMO) Measure test and Bartlett’s test of sphericity were used. Dietary patterns derived from PCA were appropriately labeled to reflect the constituents of each isolated component accounting for variation in diets. A dietary pattern, thus, refers to a distinguishable set of highly correlated food items as extracted in PCA.

Simple linear regression, controlling for age, was used to test the association between dietary pattern factor scores and child sex, weight status, school type and household SES. Sensitivity analyses showed that age used in continuous form or categorical form showed no significant changes in level of significance of associations between DPs and covariates. Thus, age was used in the categorical form in analyses. Multiple linear regression was performed to determine significant factors associated with each of the four dietary patterns. An alpha level of 0.05 was used to determine statistical significance.

## Results

### Demographic characteristics

The mean age of pupils was 12.1 years (SD = 1.5) with about 15% of them in the age 9–10 years category. Females were slightly more than males (53.6 vs. 46.4); about three-fifths of the sample were public school pupils (see Table 1).

**Table 1:** Demographic characteristics of School Age Children in Ghana who participated in study (*N* = 487)

Characteristic	Frequency	Percent
Age group		
9–10 years	73	15
11–12 years	213	43.7
13–15 years	201	41.3
School category		
Private	189	38.8
Public	298	61.2
Sex		
Male	226	46.4
Female	261	53.6
Household Socio-economic status^a^		
Poor	199	40.9
Middle	187	38.4
Rich	101	20.7
BMI status		
Overweight/Obese	86	17.7
Non-Overweight/obese	401	82.3

^a^tertiles of wealth index computed using: (ownership of car/ vehicle, house, television, refrigerator, gas/electric cooker, DVD/VCR/video player, air conditioner, and satellite dish) poor = lowest 40%; middle = middle 40%; Rich = top 20%

### Emerging dietary patterns

Results of PCA yielded a KMO = 0.85 which is meritorious (Field, 2009). Additionally, the items used had sufficiently large correlations for PCA (Bartlett’s test of sphericity = χ2 (91) = 1328.16, *p* < 0.001). Furthermore, the 14 items used in the PCA showed good consistency (Cronbach’s alpha = .78). Number of components decided upon was informed by an examination of the scree plot, which showed an inflection at the fifth component. PCA identified four dietary patterns that explained 53.2% of the variance in diets of respondents (see Table 2). The label assigned to components was based on the items with high loadings within each component as well as interpretability of factors. These included: (1) Energy dense; (2) starchy roots and vegetable/ ‘typically Ghanaian’; (3) cereal-grain and poultry/ ‘modern Ghanaian’; and (4) ‘fish and seafood-based’ diets. Table 3 shows the various food items and the truncated components solution.

**Table 2:** Dietary patterns of school age children determined using principal component analysis

Component		% of variance	Label for dietary pattern
Component 1	Sugar-sweetened beverages,	21.6	Energy dense pattern
	Fried foods		
	Processed meats		
	Spreads and toppings		
	Fruits and fruits juices		
	Cocoa beverages and dairy products		
	High calorie snacks		
Component 2	Meats (all non-poultry, non-sea food)	13.7	Starchy roots and vegetables/ typically Ghanaian
	starchy roots and tubers		
	Vegetables*		
	Legumes		
	Soups		
Component 3	Cereals/grains	10.4	Cereal-grain and poultry/ modern Ghanaian
	Poultry and eggs		
	Vegetables*		
	Soups		
Component 4	Fish and sea foods	7.4	Fish and sea food

Note: ‘wewre’ refers to sun-dried wild melon seeds used for soups or stews

*excludes tomatoes, peppers and onions since they are basic ingredients in every Ghanaian hot meal

**Table 3:** Sub-food group items and their truncated components solution

Food items	DP 1	DP 2	DP 3	DP 4	h^2^
Sugar sweetened beverages (SSB)	*0.680*	0.173	−0.090	− 0.102	0.512
High calorie snacks (including sweets & pastries)	*0.680*	0.270	0.042	− 0.005	.538
Cocoa beverages, milk and dairy	*0.638*	−0.045	0	−0.203	0.450
Processed meat	*0.592*	−0.012	0.160	0.201	0.416
Fruits and fruit juices	*0.592*	0.365	0.066	−0.056	0.491
Spreads and toppings	*0.585*	0.189	0.136	0.192	0.434
Fried foods	*0.579*	0.255	0.377	0.041	0.544
Legumes (pulses and nuts)	0.206	*0.659*	−0.031	−0.016	0.477
Meat (non-poultry)	0.117	*0.658*	0.123	0.084	0.468
Starchy roots and plantains	0.075	*0.645*	0.045	−0.056	0.427
Vegetables and soups	0.136	*0.533*	*0.409*	0.018	0.471
Cereals	−0.051	0.081	*0.854*	−0.080	0.745
Poultry and eggs	*0.483*	0.101	*0.584*	0.047	0.586
Fish and sea food	−0.014	−0.006	−0.058	*0.937*	0.882

H^2^ refers to communality; italicised entries are items contributing significantly to each PCA component

### Associations between major dietary patterns and pupils characteristics

Table 3 presents the four major dietary patterns emerging from the survey of basic school pupils age 9–15 years in urban Ghana. The first pattern “Energy dense” diet explaining 21.6% of the variation in diets of pupils was associated with higher household SES [F(2, 482) = 13.182, *p* < 0.001], child overweight [F(2, 482) = 5.185, *p* < 0.01] and private school attendance [F(2, 482) = 7.183, *p* = 0.001]. Consumption of energy dense diets was not significantly associated with sex or age of child in bivariate analyses. After controlling for child age, sex, school type and household SES, the association between consumption of energy dense diet and child overweight status remained significant [F(5, 479) = 6.868, *p* < 0.001]. Details of bivariate and multivariate linear regressions of dietary patterns and background factors are shown in Tables 4 and 5 respectively.

**Table 4:** Bivariate tests of associations between emerging dietary patterns and background characteristics

	Energy-dense			Starchy roots &vegetable			Cereal-grains & poultry			Fish & seafood		
Variables	*B*	*SE B*	*β*	*B*	*SE B*	*β*	*B*	*SE B*	*β*	*B*	*SE B*	*β*
Age	−0.074	0.064	−0.052	0.203	0.064	0.143^**^	−0.094	0.064	−0.067	0.043	0.065	0.030
Sex (female)	0.106	0.091	0.053	0.002	0.090	0.001	0.103	0.091	0.052	−0.114	0.091	−0.057
Private school	0.343	0.095	0.167^***^	−0.364	0.094	−0.178^***^	− 0.191	0.096	− 0.093*	0.009	0.096	0.004
Overweight	0.355	0.118	0.136^**^	−0.251	0.118	−0.096^*^	− 0.113	0.119	− 0.043	0.081	0.119	0.031
Household SES^a^	−0.293	0.059	−0.222	0.159	0.059	0.121^**^	0.090	0.060	0.069	0.014	0.06	0.010

*B* beta coefficient*, β* standardized beta coefficient

*p < .05

**p < .01

***p < .001

^a^from rich towards poor SES groups; associations adjusted for age only

**Table 5:** Multivariate analyses of background factors associated with emerging dietary patterns

	Energy-dense			Starchy roots &vegetable			Cereal-grains & poultry			Fish & seafood		
Variables	*B*	*SE B*	*β*	*B*	*SE B*	*β*	*B*	*SE B*	*β*	*B*	*SE B*	*β*
Age	−0.021	0.065	−0.015	0.138	0.065	0.097^*^	−0.13	0.067	−0.091	0.049	0.067	0.035
Sex	0.046	0.09	0.023	0.045	0.09	0.023	0.127	0.092	0.064	−0.126	0.093	0.063
Private school	0.153	0.104	0.075	−0.301	0.105	−0.147^**^	− 0.154	0.107	− 0.075	0.018	0.107	0.009
Overweight	0.246	0.119	0.094^*^	−0.189	0.119	−0.072	−0.099	0.122	−0.038	0.112	0.123	0.043
Household SES^a^	−0.229	0.065	−0.174^***^	0.064	0.065	0.049	0.046	0.067	0.035	0.022	0.067	0.017
*R* ^*2*^			0.067			0.058			0.008			0.006
*F*			6.868			5.868			1.783			0.578

*B* Beta coefficient*, β* standardized beta coefficient

*p < .05

**p < .01

***p < .001

^a^from rich towards poor SES groups

The second pattern, “starchy roots &vegetable-based diet”, which is ‘typically Ghanaian’, explained 13.7% of variation in pupil diets and was negatively associated with overweight status [F(2, 482) = 7.369, *p* = 0.001]. It was also associated with older age groups [F(1, 483) = 10.091, *p* = 0.002], public school attendance [F(2, 482) = 12.702, *p* < 0.001] and lower household SES [F(2, 482) = 8.725, *p* < 0.001] categories. After controlling for age, sex, school type and household SES, consumption of the ‘starchy root & vegetable diet’ was no longer associated with child weight status. However, the starchy roots &vegetable-based diet was significantly associated with public school attendance and older age groups after controlling for household SES, age and weight status [F(5, 479) = 5.868, *p* < 0.001].

The third DP was negatively associated with private school attendance at the bivariate stage [F (2, 482) =3.059, *p* < 0.05]. Both the third and fourth identified dietary patterns were not significantly associated with any of the child background characteristics examined in this study in multivariate analyses.

A multiple logistic regression examining the relationship between child weight status and the four identified dietary patterns controlling for age, sex, school type and household SES showed a positive association between energy dense pattern and child overweight (AOR = 1.34, 95%CI = 1.04–1.70). Male sex was significantly associated with reduced odds of being overweight (AOR = 0.41, 95%CI = 0.24–0.69) after controlling for age, school type, household SES and dietary pattern. Childhood overweight was not significantly associated with any of the other three dietary patterns.

## Discussion

This study aimed to describe the dietary patterns emerging from food consumption data of school age children in urban Ghana. Four main food consumption patterns were identified: energy dense; starchy roots and vegetables; grain-based and poultry; and fish/seafoods. Both the healthy and unhealthy food patterns were associated with child age and household socioeconomic status.

### Major dietary patterns emerging from consumption data

The first major dietary pattern (DP), “energy dense” identified in this study was characterized by the consumption of sugar sweetened beverages, candies, ice creams, fried foods, processed meats, spreads, toppings, and snacks like cakes, pies, doughnuts and other savory foods. This diet pattern typically referred to as the “westernized diet” has been identified in other non-western societies including Ghana, probably due to industrialization and global food market expansion [3, 45, 46]. This DP has also been seen in almost all studies that sought to examine dietary patterns among children and adolescents elsewhere [47–49]. Food items associated with the energy dense pattern have been reported elsewhere, although sometimes split into different but related components. For instance, Pala et al. [47] identified two energy dense patterns labeled “snacking” and “sweet and fat” which together explained most (16%) of the variability in the diets of European children. Also, Correa et al. [50] described a similar pattern as “Brazilian industrialized”. Findings of this study agrees with the body of evidence linking energy-dense DP consumption and overweight/obesity among children [48, 51, 52]. However, Pala et al. [47] found no association between energy dense DPs and overweight status among European children in a longitudinal study. Energy dense DP, was significantly associated with higher SES and its’ proxy, private schools; the relationship remained significant after controlling for all background characteristics. The association between higher SES groups and ‘unhealthy’ dietary behaviours has been typically reported in Ghana and other developing countries, but contrary to developed country situations. For instance, Craig et al. [48] reported energy dense DP among lower SES groups while healthier DPs were associated with children belonging to higher SES groups. This disparity between developing and developed countries regarding the link between SES and health outcomes is well documented as a function of countries’ overall GDP [53] and will eventually shift towards lower SES groups over time. Nevertheless, the negative effects of energy dense diets on child weight status in Ghana, irrespective of SES, suggests need for interventions to control consumption of energy dense DPs.

The second (starchy roots and vegetable) and third (cereal-grain and poultry) DPs identified in this study both include the consumption of staples typically available in Ghana. However, there are differences in consumption due to the changing diets of Ghanaians as documented by Goody and Goody [9]. Foods in the third DP are likely more ‘prestigious’ and less labour intensive compared to those in the third DP. Additionally, foods in the grain-cereal and poultry DP are increasingly made available in school canteens and sold by food vendors as they are generally more appealing to children. Meanwhile, foods in the starchy roots and vegetables’ DP are generally more affordable and can be accessed easily from vendors outside of the school. It is therefore not surprising that the consumption of the second DP was associated with lower SES groups, older age of child, and public-school attendance. Considering that only 5% of pupils in a previous study brought food daily from home [54], sourcing of food from the school and its’ environs remains inevitable. Also, the likelihood that parents will pack traditional meals (like *kenkey, banku, kokonte, gari*, *eba*, rice ball,^3^fufu), which are served with soups and vegetables is low due to the inconvenience (possible spills and requires skill to eat without stains) and low prestige associated with traditional foods. The soups and stews served with these typically Ghanaian foods are also highly perishable and there are food safety concerns about packing it for children. Consequently, younger aged children will consume more foods in the third DP, grains and chicken compared to the older children who will may patronize local foods they have developed preference for at school. While this study did not assess the usual sources of staple foods, anecdotal evidence suggests that these typically Ghanaian foods are more likely to be consumed at home with family during supper on school days or anytime on weekends. Thus, children living in homes that do not cook meals frequently, will generally record lower consumption of DP 2 and a higher consumption of DP 3.

Although studies among European children found a protective effect of DP based on whole grains, legumes and vegetables [47] on weight, this study did not find significant associations between the third DP and SES, age, school type and weight status of children. This finding must be interpreted with caution since the relationship is likely influenced by nature of data collected. Our study questionnaire did not discriminate between whole and refined cereal/grain consumed by the children. Again, the poultry consumed in this DP with cereals is most likely fried and or stewed, considering how grains-based meals are typically served (both at home and school) or sold in Ghana.

The last DP, “fish and sea foods” explained 7.4% of the variation in the diets of the children surveyed and was not associated with any child characteristic. While it is not normal for fish and seafoods alone to be consumed in any diet, this sub-food group recorded the highest communality (.882) compared to the remaining 15 items used in PCA, suggestive of relevance. Craig and colleagues [48] identified “fish and sauce” DP among children 5–11 years old in Scotland. The consumption of fish as part of a healthy diet is beneficial [55]. Fish is widely consumed in Ghana [8, 56] and most often served whole (with head and bones) irrespective of cooking method. It is possible that the skill required to eliminate bones as well as other factors may influence both parent and children’s’ from choice of fish compared to ‘easier to consume’ options like eggs or poultry. An assessment of fish consumption by school-age children may provide evidence required to understand its inclusion in diets and how it relates with weight status.

To our knowledge, this is the first study to identify dietary patterns in the diets of school-age children in Ghana. We agree that this study is cross-sectional and thus, causality cannot be assumed for the observed association between energy dense dietary pattern consumption and child overweight/obese status. Nevertheless, the identification of dietary patterns similar to those found in other studies is a contribution to knowledge regarding the public health goal of identifying optimal dietary patterns across settings.

A limitation of this study is the inability of data collected on cereal grains to distinguish between refined and unrefined grains consumed. The researchers felt that it would be nearly impossible for children to provide this detail for all grains eaten in and out of the home. This anticipated difficulty is also due to the possibility of these grains to be masked when cooked in a composite meal like “jollof rice” or ‘^4^Wakye’, which are popularly consumed forms among children. Also, grains available in Ghana are mostly whole grain with the exception of rice and maize which have polished versions. Nevertheless, we believe that this had no significant influence on the major dietary patterns identified among the children. Secondly, our classification of households based on possession of 8 assets may have oversimplified or poorly ranked households. Nevertheless, the use of household assets in ranking households in this setting is reliable [42]. Lastly, we acknowledge that no single dietary assessment method, including the FFQ, is able to perfectly assess dietary exposure. Nevertheless, the FFQ when specifically designed for target groups, remains the tool of choice in measuring usual intakes of populations [57].

## Conclusions

This study identified four dietary patterns; with the unhealthy pattern significantly associated with overweight/obesity, higher household SES and older age groups. This has implications for later health outcomes because dietary habits are likely to worsen in older adolescence [26]. Adolescence is a critical period for establishing lifetime dietary patterns and if a significant component of their diet are energy-dense-low nutrient options, it imposes extra risk of poor nutrition and adverse health outcomes. The evidence shows the existence of both traditionally available healthy foods as well as foods typical of ‘western among Ghanaian school age children (9–15 years). This provides an opportunity for promoting more healthful food choices especially among middle and higher SES groups during health promotions.

## Additional files

Additional file 1: 7-day Food Frequency Questionnaire. (DOCX 21 kb)

Additional file 2: Food items defined under specific sub-food groups used in the PCA. (DOCX 15 kb)

## Abbreviations

BMA: BMI for Age and Sex
DP: Dietary pattern
FFQ: Food frequency questionnaire
JHS: Junior High School
PCA: Principal component analysis
SES: Socio Economic Status

## Footnotes

1 Kenkey is boiled fermented corn dough balls wrapped in corn husks; can be mashed and sweetened with sugar and enjoyed alone or with bread, or balls eaten with hot sauce, soup or stew

2 Banku is made from fermented corn dough mixed with fermented cassava dough. The paste is stirred over heat to solidify, made into balls and served with pepper sauce, soups or stews

3 Fufu is cooked starchy roots or tubers that have been pounded into a smooth paste; usually eaten with soup

4 Wakye is rice and beans cooked together
