# Supplementary material for: Dietary patterns and associated risk factors among school age children in urban Ghana
Source: BMC Nutr. 2018 May 10;4:22. doi: 10.1186/s40795-018-0230-2 (PMC7050789; doi:10.1186/s40795-018-0230-2)
Supplement: Supplementary file 1 — 7-day Food Frequency Questionnaire. (DOCX 21 kb) [file 40795_2018_230_MOESM1_ESM.docx]

Additional file 1 7-Day Food Frequency Questionnaire

**Please tell me whether you ate any of the following foods during the past one week and how often?**

| **CODE** | **SWEETENED DRINKS AND FRUIT JUICES** | | | **Past One week**  **1=Yes**  **2=No** | **NO. OF TIMES PER WEEK** | | **WHERE DID YOU GET YOUR FOOD FROM?**  **(*Please write*)**  **0=Not Applicable**  **1=Home**  **2=School vender**  **3=Street food** |
| --- | --- | --- | --- | --- | --- | --- | --- |
| SWTD | Sweetened drinks (Tampico, Kalyppo, Refresh) | | |  |  | |  |
| FRUJ | Fruit juices (Pure Heaven, Ceres, Nourisher) | | |  |  | |  |
| MALD | Malt drinks (Malta Guinness, Vitamalt, Amstel) | | |  |  | |  |
| MIN | Minerals (eg. Fanta, Sprite, Coca Cola) | | |  |  | |  |
| LOCD | Local drinks (asana, shitor da, etc.) | | |  |  | |  |
| OTHER |  | | |  |  | |  |
| **COCOA, MILK AND DAIRY PRODUCTS** | | | | | | | |
| MILK | Milk (evaporated/powdered/fresh) | | |  |  | |  |
| MILD | Milk drinks (Countre Milk, Milko, miksi, cowbell) | | |  |  | |  |
| YOGT | Yogurt | | |  |  | |  |
| CHES | Cheese / Wagashi | | |  |  | |  |
| MILO | Milo/ cocoa beverages | | |  |  | |  |
| **BREADS, BISCUITS AND PASTRIES** | | | | | | | |
| BRED | Bread (sugar or tea bread) | | |  |  | |  |
| BISC | Biscuits / cookies/ crackers | | |  |  | |  |
| CAKE | Cakes | | |  |  | |  |
| PAST | Other pastries (pies, baked chips, bans etc.) | | |  |  | |  |
| PIZA | Pizzas | | |  |  | |  |
| BBP1 | Other: | | |  |  | |  |
| **FRIED FOODS** | | | | | | | |
| BOFR | Bofrot / Donuts | | |  |  | |  |
| KOSE | Akara / Koose | | |  |  | |  |
| FRIET | Fried plantain / Kelewele/yam/ sweet potatoes/ | | |  |  | |  |
| FRYPO | Fried potato/ chips | | |  |  | |  |
| PASTR | Flour chips / Pastries | | |  |  | |  |
| FREEG | Fried eggs | | |  |  | |  |
| FRYCH | Fried chicken and poultry | | |  |  | |  |
| FRYMT | Fried meat | | |  |  | |  |
| FRYRC | Fried rice/ jollof rice/ braised rice | | |  |  | |  |
| FFO1 | Other: | | |  |  | |  |
|  |  | | |  |  | |  |
| **CODE** | | **PROTEIN FOODS** | **Past One week:1=Yes**  **2=No** | | | **NO. OF TIMES PER WEEK** |  |
| MEAT | | Meat (pork, beef, mutton, bush meat) |  | | |  |  |
| POUL | | Poultry (chicken, duck, turkey, birds) |  | | |  |  |
| KHEB | | Kebab |  | | |  |  |
| CORNB | | Corned beef / luncheon meat |  | | |  |  |
| SAUS | | Sausage / Bacon |  | | |  |  |
| EGGS | | Eggs |  | | |  |  |
| BURG | | Burgers |  | | |  |  |
| FISH | | Fish |  | | |  |  |
| PFO1 | | Other: |  | | |  |  |
| **SPREADS AND TOPPINGS** | | | | | | |  |
| MAGR | | Margarine / Butter |  | | |  |  |
| JAM | | Jam |  | | |  |  |
| GPAST | | Groundnut (peanut) paste |  | | |  |  |
| CHOS | | Chocolate spread |  | | |  |  |
| SALC | | Salad cream |  | | |  |  |
| MAYO | | Mayonnaise |  | | |  |  |
| SPRO1 | | Other: |  | | |  |  |
| **FRUITS** | | | | | | |  |
| CITR | | Citrus (Orange, Tangerine, Grape fruit, star fruit) |  | | |  |  |
| PINE | | Pineapple |  | | |  |  |
| WATM | | Water melon |  | | |  |  |
| MANG | | Mango |  | | |  |  |
| BANN | | Banana |  | | |  |  |
| PEAR | | Avocado pear |  | | |  |  |
| PAWP | | Pawpaw |  | | |  |  |
| GUAV | | Guava |  | | |  |  |
| APPL | | Apple |  | | |  |  |
| SUGC | | Sugar cane |  | | |  |  |
| FRO1 | | Other: |  | | |  |  |
| **VEGETABLES** | | | | | | |  |
| KONT | | Kontomire |  | | |  |  |
| GREL | | Other dark green leafy vegetables |  | | |  |  |
| CARP | | Carrots |  | | |  |  |
| OKRO | | Okra |  | | |  |  |
| GEGG | | Garden eggs/ Aubergines |  | | |  |  |
| VEGO1 | | Other: |  | | |  |  |
| VEGO2 | | Others |  | | |  |  |
| **Soups** | | | | | | |  |
| PALMS | | Palm soup |  | | |  |  |
| GNUTS | | Groundnut soup |  | | |  |  |
| KONTS | | Kontomire soup |  | | |  |  |
| OTHER S | |  |  | | |  |  |
| **OTHER HIGH FAT/CALORIE FOODS** | | | | | | |  |
| CHOCO | | Chocolate |  | | |  |  |
| TOFFE | | Toffees/candies/lollipops |  | | |  |  |
| NUTS | | Nuts (groundnuts/peanuts, cashew, tiger nuts) |  | | |  |  |
| SUGA | | Sugar |  | | |  |  |
| ICECR | | Ice cream |  | | |  |  |
| POPC | | Popcorn |  | | |  |  |
| **STAPLE FOODS** | | | | | | |  |
| MAIZE | | Maize based foods (eg. Kenkey, Aple, Banku, TZ) |  | | |  |  |
| CEREAL | | Other cereals (eg. Rice, millet, sorghum, wheat) |  | | |  |  |
| TUBER | | Root and tubers (eg. Fufu, kokonte, cassava, gari) |  | | |  |  |
| LEGUME | | Legumes (eg.cowpeas, bambara, soya beans, melon seeds, wrewre, kidney beans,) |  | | |  |  |
| WAKYE | | Waakye |  | | |  |  |
