# Supplementary material for: Dietary patterns and associated risk factors among school age children in urban Ghana
Source: BMC Nutr. 2018 May 10;4:22. doi: 10.1186/s40795-018-0230-2 (PMC7050789; doi:10.1186/s40795-018-0230-2)
Supplement: Supplementary file 2 — Food items defined under specific sub-food groups used in the PCA. (DOCX 15 kb) [file 40795_2018_230_MOESM2_ESM.docx]

Additional file 2 Food items defined under specific sub-food groups used in the PCA

| Sub-food group | Constituent food items |
| --- | --- |
| Sugar-sweetened beverages | Sugar-sweetened drinks (Tampico, Kalyppo, Refresh, etc)  Malt drinks (Malta Guinness, Vitamalt, Amstel, etc.)  Minerals/ carbonated drinks (eg. Fanta, Sprite, Coca Cola, 7-up, etc.)  Local drinks (*asana* (malted maize drink), *shitor da* (ginger drink), etc.)  Fruit drinks |
| Fried foods | Akara / Koose (made from cowpea), Bofrot / Donuts  Fried plantain / Kelewele/yam/ sweet potatoes/  Fried potato/ chips  Deep fried Flour chips / Pastries  Fried eggs  Fried chicken and poultry  Fried meat  Fried rice/ jollof rice/ braised rice |
| Processed meats | Sausages, burgers, Corned beef, luncheon meat, beef pates, hotdogs, etc. |
| Spreads and toppings | Margarine, jam, mayonnaise, salad cream, chocolate and other spreads |
| Fruits and fruits juices | Oranges, pawpaw, pineapples, bananas, avocado pears, star fruit, water melons, grapes, grape fruit, guava, etc.  Fruit juices (Pure Heaven, Ceres, Nourisher) |
| Cocoa beverages and dairy products | Milk (liquid and powdered), cheese, *wagashi* (local cheese), yoghurts, cocoa and flavored milk drinks (e.g. Milo, country milk, etc.) |
| High calorie snacks | Cakes, pies, biscuits, doughnuts, Toffees/candies/lollipops, sugar, popcorns, ice creams, Chocolates, other pastries and packaged snacks |
| Meats | pork, rabbit, grass cutter, beef, mutton, bush meat, goat meat, all non-poultry non-sea food |
| starchy roots and tubers | (yams, cassava, plantain, cocoyam, taro, sweet potatoes) |
| Vegetables | green leafy (*Kontomire*, *aleefu*, *ademe, ayoyo*, and  other vegetables: okra, carrots, cabbages, cauliflower, egg plants, etc. |
| Legumes | Pulses: includes black eyed pea, melon seeds, red beans, Bambara beans, *wrewre*, all other beans and nuts (groundnuts, cashew nuts, etc.) |
| Soups | palmnut, groundnut soup, green vegetable soup, light soup, mixed vegetable soup, ‘*wrewre* soup, etc |
| Cereals-grains | maize, wheat, rice, oats, millet, guinea corn, sorghum, etc and their products |
| Poultry and eggs | Chicken, turkey, duck, other birds and eggs |
| Fish and sea foods | Fish, crabs, shrimps, oysters, clams, etc. |
